# Smoking, GAD65 autoimmunity, genetic susceptibility to type 1 diabetes and incident adult-onset diabetes in the EPIC-InterAct case–cohort study

**DOI:** 10.1007/s44357-026-00003-9

**Published:** 2026-04-16

**Authors:** Emmy Keysendal, Sofia Carlsson, Anna-Maria Lampousi, Christiane S. Hampe, José M. Huerta, Nicola Kerrison, Peter M. Nilsson, Valeria Pala, Matthias B. Schulze, Stephen J. Sharp, Olov Rolandsson, Nicholas J. Wareham

**Affiliations:** 1 Institute of Environmental Medicine, Karolinska Institutet, Stockholm, Sweden; 2 Department of Medicine, University of Washington School of Medicine, Seattle, WA USA; 3 Department of Epidemiology, Murcia Regional Health Council-IMIB, Murcia, Spain; 4 CIBER Epidemiología y Salud Pública (CIBERESP), Madrid, Spain; 5 Medical Research Council Epidemiology Unit, Institute of Metabolic Science, University of Cambridge, Cambridge, UK; 6 Department of Clinical Sciences, Clinical Research Center, Skåne University Hospital, Lund University, Malmö, Sweden; 7 Epidemiology and Prevention Unit, Fondazione IRCCS Istituto Nazionale Dei Tumori, Milan, Italy; 8 Department of Molecular Epidemiology, German Institute of Human Nutrition Potsdam-Rehbruecke, Nuthetal, Germany; 9 Institute of Nutritional Science, University of Potsdam, Potsdam, Germany; 10 Department of Public Health and Clinical Medicine, Family Medicine, Umeå University, Umeå, Sweden

**Keywords:** Diabetes, GAD65Ab, Genetic susceptibility, Interaction, Smoking

## Abstract

**Aims/hypothesis:**

Smoking increases the risk of type 2 diabetes, but its role in autoimmune diabetes remains unclear. We investigated whether smoking is associated with being GAD65 autoantibody (GAD65Ab) positive or the risk of progressing to diabetes. We also assessed its interaction with genetic susceptibility to type 1 diabetes.

**Methods:**

We used data from the EPIC-InterAct case–cohort study including 11,161 incident cases of adult-onset diabetes and 14,922 subcohort participants. Logistic regression was used to estimate odds ratios (ORs) for being GAD65Ab positive at baseline by smoking status. Hazard ratios (HRs) of diabetes by baseline smoking and GAD65Ab status were estimated using Prentice-weighted Cox regression. Two- and three-way interactions between smoking, GAD65Ab status and a type 1 diabetes genetic risk score (T1D-GRS) were assessed by attributable proportion due to interaction (AP).

**Results:**

There was no evidence that smoking was associated with being GAD65Ab positive (OR 1.00, 95% CI 0.82, 1.22 current vs never smoking), but among GAD65Ab-positive participants current smokers were more likely to progress to diabetes than never smokers (HR 1.40, 95% CI 1.03, 1.90) and there was an interaction between being GAD65Ab positive and heavy smoking for diabetes risk (AP 0.32, 95% CI 0.15, 0.49 ≥ 15 pack-years vs never smoking). Three-way interaction between heavy smoking, being GAD65Ab positive and the highest T1D-GRS tertile was also observed (combined HR 7.30, 95% CI 5.17, 10.31; AP 0.46, 95% CI 0.23, 0.70).

**Conclusions/interpretation:**

While smoking may not be associated with being GAD65Ab positive, it may increase the risk of progressing to diabetes in GAD65Ab-positive individuals and in those with genetic susceptibility to type 1 diabetes.

**Graphical Abstract:**

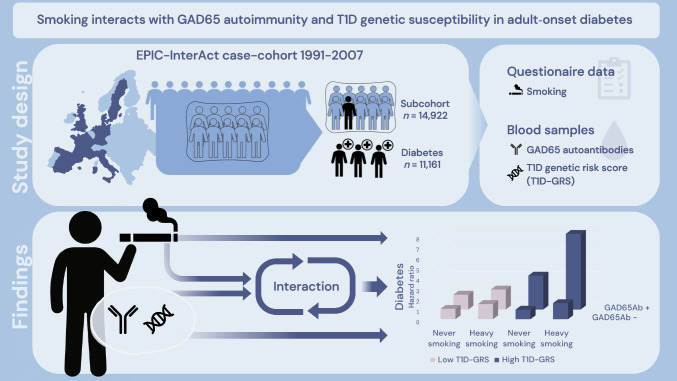

**Supplementary Information:**

The online version contains supplementary material available at 10.1007/s44357-026-00003-9.

## Introduction


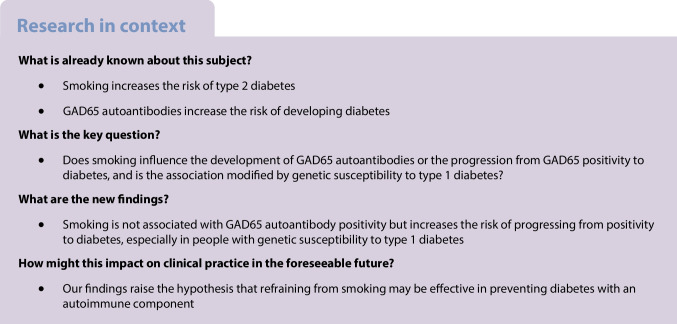


The pathogenesis of autoimmune diabetes involves an autoimmune response, causing destruction of the insulin-producing beta cells and insulin deficiency [1]. The presence of antibodies targeting the 65 kDa isoform of the pancreatic enzyme glutamic acid decarboxylase (GAD65Ab) is a marker of autoimmunity and is linked to an increased risk of developing diabetes in adulthood [2]. Lifestyle or environmental factors may promote disease progression and transition from GAD65 autoimmunity to diabetes in adults [3]; however, studies defining such risk factors are limited.

Smoking is known to increase the risk of type 2 diabetes [4]. The underlying mechanism behind this association is hypothesised to involve nicotine-induced reductions in insulin sensitivity [5]. For autoimmune diabetes in adults, smoking has been linked to a higher incidence of both latent autoimmune diabetes in adults (LADA) [6] and type 1 diabetes in previous Scandinavian studies [7]. For LADA, smoking has been shown to interact with type 1 diabetes-associated human leukocyte antigen (HLA) genotypes to increase diabetes risk [6]. In contrast, maternal smoking during pregnancy has been associated with a lower risk of childhood-onset type 1 diabetes [8], an effect thought to be driven by the immunosuppressive properties of nicotine [9]. Smoking has also been positively associated with the development of other autoimmune diseases, including rheumatoid arthritis [10, 11] and systemic lupus erythematosus [12]. In rheumatoid arthritis, smoking is thought to promote autoantibody development through citrullination [13]. Similar mechanisms have been suggested for GAD65Ab in autoimmune diabetes [14]. However, it remains unclear whether smoking is related to the development of type 1 diabetes-associated autoantibodies, such as GAD65Ab, or acts as a ‘second hit’ to accelerate disease development in autoantibody-positive individuals. Further investigation of the potential effect of smoking on the development of autoimmunity and autoimmune disease is therefore warranted.

We aimed to clarify the impact of smoking on the development of autoimmune diabetes in adults. Specifically, we examined whether smoking was associated with being GAD65Ab positive or the risk of diabetes progression in GAD65Ab-positive individuals. Additionally, we investigated potential interaction effects between smoking, being GAD65Ab positive and genetic susceptibility to type 1 diabetes on subsequent diabetes risk.

## Methods

### Study population

This study used data from EPIC-InterAct, a prospective case–cohort study within the EPIC (European Prospective Investigation into Cancer and Nutrition) cohort [15]. Participants were recruited across 26 centres in eight European countries: Denmark, France, Germany, Italy, the Netherlands, Spain, Sweden and the UK. Enrolment occurred between 1991 and 1998 with collection of blood samples and questionnaire and anthropometric data. Individuals without stored blood or diabetes status information were excluded, leaving 340,234 eligible participants who were followed until diabetes diagnosis, death or 31 December 2007. Information on ethnicity was not collected, and the cohort is largely representative of adults of European origin from diverse regions and socioeconomic backgrounds. The cohort includes both women and men, but sex was not considered in the study design. The study accrued 3.99 million person-years of follow-up, and a centre-stratified subcohort was created including 16,835 participants (4.9% of the full cohort) randomly selected from the full cohort. The case–cohort study consisted of 16,154 subcohort participants without prevalent diabetes or unknown diabetes status and 12,403 verified incident cases, of which 778 were overlapping with the subcohort. Informed consent was obtained from all participants [16]. The study was approved by the local ethics committee in each EPIC country and the Internal Review Board of the International Agency for Research on Cancer [15].

In the current study 1242 cases and 1232 subcohort participants were excluded from analysis, 576 cases and 535 subcohort participants for missing information on GAD65Ab or smoking status, and 666 cases and 697 subcohort participants due to missing covariate data on BMI, education level, physical activity level or meat, coffee or alcohol intake. This left 11,161 cases and 14,922 subcohort individuals eligible for analysis (ESM Fig. 1). Smoking intensity or duration was available for 9101 cases and 12,553 subcohort participants. In analyses of genetic data, 9124 cases and 12,645 subcohort participants remained after excluding 4742 individuals with missing genetic information.

### GAD65Ab measurements

Blood samples with extracted blood plasma were collected at enrolment and stored at −196 °C in liquid nitrogen, except in Umeå where samples were stored at −80 °C [15]. Baseline GAD65Ab levels were analysed using a radioligand binding assay [2] and expressed in relative units based on the WHO standard [17]. The assay showed 70% sensitivity and 98% specificity for GAD65Ab in the International Combined Autoantibody Workshop [18]. In addition, the threshold for positivity was set at ≥65 units/ml, which generated 99% assay specificity and 85% sensitivity [2]. As in previous studies on this population, the median GAD65Ab level among positive individuals (167.5 units/ml) was used as the cut-off for high GAD65Ab levels [2].

### Genotyping

DNA was extracted and quantified from blood [15], and genotyped using two genotyping platforms: the Illumina 660W-Quad BeadChip (Illumina, San Diego, CA, USA) at the Wellcome Trust Sanger Institute and the Illumina core-exome 12v1 and 24v1 arrays at Cambridge Genomic Services in the Department of Pathology at the University of Cambridge [19]. In total, 20,645 participants had genetic data available for inclusion in genetic analysis.

### Genetic risk score for type 1 diabetes

We recreated a type 1 diabetes genetic risk score (T1D-GRS, sometimes referred to as T1D-GRS1) based on 30 genetic variants previously used in EPIC-InterAct [2, 20]. HLA DR haplotypes (HLA-DRB1*03:01 and HLA-DRB1*04 with DQB1*03:02) were assigned as dichotomised variables using rs2187668 and rs7454108 genotypes. Genotypes were assigned as hard-call dosages with a threshold of 0.5. The GRS was calculated as the sum of risk-increasing alleles (0, 1 or 2 for SNPs and 0 or 1 for HLA DR haplotypes), each weighted by its log odds ratio (OR), divided by the total number of alleles considered [20]. The GRS was analysed continuously and in tertiles based on the subcohort distribution.

### Diabetes ascertainment

Incident diabetes cases were ascertained based on self-reported data, hospital admissions, mortality records or information retrieved from healthcare or drug registers. At least two sources had to report a diagnosis for a case to be verified, except in Sweden and Denmark where cases were derived from either local and national diabetes registers or pharmaceutical registers as the only sources [15]. Some diabetes cases may meet the common LADA criteria, including being GAD65Ab positive, age at onset > 35 years, and no insulin treatment in the first 6–12 months after diagnosis [21]. As GAD65Ab levels were measured only at baseline, not at diagnosis, we could not confirm autoimmune status at onset and, therefore, cases could not fulfil the clinical definition of LADA. Accordingly, we refer to the outcome as adult-onset diabetes.

### Smoking assessment

Baseline smoking information was collected through lifestyle questionnaires across all centres, including smoking status (never, former, current), age at initiation and cessation, type of tobacco used (cigarettes, cigars, pipes) and smoking intensity (number of cigarettes smoked per day). Pack-years of cigarette smoking were calculated as (lifetime smoking intensity/20) × duration of smoking in years, assuming 20 cigarettes per pack. Smoking intensity and pack-years among ever smokers were analysed continuously (per 5 cigarettes/day and per 5 pack-years) and categorically (never, < 20 cigarettes/day or ≥ 20 cigarettes/day, and never, < 15 pack-years or ≥ 15 pack-years), using cut-offs consistent with those applied in recent studies of diabetes [22].

### Baseline covariate assessment

Demographic and lifestyle information including age, sex, education level (none, primary, technical/professional, secondary, tertiary), physical activity level (inactive, moderately active, active), diet and alcohol consumption (g/day) was collected at baseline through questionnaires. Self-reported data on physical activity generated an index, validated within the InterAct project [23]. Anthropometric data including height (m), weight (kg) and waist circumference (cm) were measured at health examinations across centres using similar protocols, except in France and Oxford where data were partly or fully self-reported. BMI was calculated as weight divided by height squared (kg/m^2^). Country-specific diet questionnaires, including intake of coffee and meat, were either self-administered or facilitated by interviewers [16].

### Statistical analysis

Baseline characteristics of GAD65Ab-positive and GAD65Ab-negative participants were compared within incident cases and the subcohort using unpaired *t* tests (means and SDs) and Kruskal–Wallis tests (medians and IQRs). Categorical variables were summarised as percentages and compared using χ^2^ tests. Distributions of GAD65Ab concentrations were visualised graphically (ESM Fig. 2).

#### Cross-sectional analyses

Logistic regression was used to estimate odds ratios (ORs) and 95% CIs of being GAD65Ab positive at baseline in relation to smoking and T1D-GRS (low vs intermediate and high). Overall *p* values were obtained from Wald tests to assess the joint significance of smoking categories. Tobit regression was used to analyse the association between smoking and continuous GAD65Ab levels (truncated at 1000 units/ml).

#### Prospective analyses

Prentice-weighted Cox regression (24) was used to estimate hazard ratios (HRs) with 95% CIs for adult-onset diabetes, assessing associations with baseline GAD65Ab status, T1D-GRS and smoking status, with the last stratified by GAD65Ab status. Overall *p* values were obtained from Wald tests to assess the joint significance of smoking categories.

Additive interactions between being GAD65Ab positive (overall positivity, low GAD65Ab positive, high GAD65Ab positive) and smoking (ever, intensity and pack-years) were evaluated using attributable proportion due to interaction (AP). AP quantifies additive interaction by estimating the proportion of diabetes cases attributed to the combined exposure of smoking and being GAD65Ab positive, beyond the sum of the individual contribution of each exposure. An AP value > 0 indicates a positive additive interaction (i.e. a greater than additive joint effect), whereas an AP value < 0 indicates a negative additive interaction (i.e. a less than additive joint effect). AP was calculated as:

$$AP= \frac{{HR}_{11}-{HR}_{10}-{HR}_{01}+1}{{HR}_{11}}$$

where HR_11_ denotes the HR for double exposure (e.g. smoking and being GAD65Ab positive), and HR_01_ or HR_10_ indicates single exposure (e.g. never smoked but GAD65Ab positive) [25, 26]. For three-way interaction between being GAD65Ab positive, smoking and T1D-GRS (low/intermediate vs high), AP was calculated as:

$$AP= \frac{{HR}_{001}+{HR}_{010}-{HR}_{011 }+ {HR}_{100}-{HR}_{101}-{HR}_{110}+ {HR}_{111}-1}{{HR}_{111}}$$

which reflects the proportion of risk attributable to the combined effect of all three exposures beyond the contributions of individual exposures and any two-way interactions [26].

As a complement we assessed interaction on the multiplicative scale by including product terms between GAD65Ab status and smoking status (two-way interaction) or GAD65Ab status, smoking status and T1D-GRS (three-way interaction) in the Cox models. Multiplicative interaction is considered significant with a Wald test *p* value < 0.05, indicating heterogeneity in the effect of smoking across strata of GAD65Ab status.

Consistent with the prespecified analysis plan, comparable to a previous EPIC-InterAct study on smoking and diabetes risk [27], all models were adjusted for age (underlying timescale in Cox regression), sex, centre (stratified baseline hazard in weighted Cox regression or as a fixed effect covariate in Tobit regression), education level, physical activity level, BMI, coffee consumption, alcohol intake and meat consumption (red and processed meat). Models with T1D-GRS as the main exposure were adjusted for age, sex and centre.

### Sensitivity analyses

Because of a large proportion of missingness, additional adjustment for waist circumference (6.8% missingness) and family history of type 2 diabetes (50.7% missingness) was performed as sensitivity analysis, excluding participants with missing data. Dietary factors previously linked to diabetes incidence, namely tea consumption [28], fish intake [29] and fruit and vegetable intake [30], were also adjusted for, added separately to the main model. Furthermore, individuals with baseline HbA_1c_ ≥ 48 mmol/mol (≥ 6.5%) (*n* = 2118) and those diagnosed with diabetes within 2 years after baseline (*n* = 950) were excluded in additional sensitivity analysis, as they may have been prevalent cases with delayed diagnoses, posing a risk of reverse causation.

All analyses were performed using R 4.3.1 (R Core Team, Vienna, Austria).

## Results

### Baseline characteristics

At baseline, 3.5% (*n* = 390) of the diabetes cases and 2.0% (*n* = 299) of the subcohort participants were GAD65Ab positive. The GAD65Ab-negative cases were more likely to be male and to have a higher BMI, a larger waist circumference, greater alcohol and meat consumption and a family history of type 2 diabetes than GAD65Ab-positive diabetes cases (ESM Table 1).

### Cross-sectional analyses of smoking, T1D-GRS and GAD65Ab at baseline

There was no association between smoking and being GAD65Ab positive at baseline (Table 1). The OR for current vs never smokers was estimated at 1.00 (95% CI 0.82, 1.22) for GAD65Ab positivity and 1.02 (95% CI 0.77, 1.36) for high GAD65Ab positivity. Additionally, no association was observed between smoking and continuous levels of GAD65Ab at baseline (Table 1). A high T1D-GRS (highest vs lowest tertile) was associated with baseline GAD65Ab positivity (OR 2.42, 95% CI 1.94, 3.02) and high positivity (OR 3.39, 95% CI 2.46, 4.67) (ESM Table 2).

**Table 1:** ORs and regression coefficients (95% CIs) of baseline GAD65Ab positivity and high GAD65Ab positivity by smoking status

	GAD65Ab negative, *n*	GAD65Ab positive^a^		High GAD65Ab positive^b^		GAD65Ab concentration^c^	
		*n*	OR (95% CI)	*n*	OR (95% CI)	*n*	Regression coefficient (95% CI)
Smoking status							
Never	11,035	310	1.00 (reference)	153	1.00 (reference)	11,345	0.00 (reference)
Former	7117	179	0.94 (0.77, 1.14)	92	0.96 (0.73, 1.26)	7296	− 17.68 (− 41.08, 5.73)
Current	6569	177	1.00 (0.82, 1.22)	84	1.02 (0.77, 1.36)	6746	− 1.94 (− 26.33, 22.45)
Overall *p* value			0.715		0.849		0.294
Never	11,035	310	1.00 (reference)	153	1.00 (reference)	11,345	0.00 (reference)
Ever^d^	13,686	356	0.97 (0.82, 1.14)	176	0.99 (0.78, 1.25)	14,042	− 10.41 (− 30.47, 9.64)
Cigarettes/day							
Never	11,035	310	1.00 (reference)	153	1.00 (reference)	11,345	0.00 (reference)
< 20 cigarettes/day^d^	7193	170	0.88 (0.71, 1.09)	80	0.86 (0.63, 1.17)	7363	− 11.74 (− 34.86, 11.38)
≥ 20 cigarettes/day^d^	2317	63	1.12 (0.82, 1.51)	31	1.23 (0.80, 1.90)	2380	− 22.60 (− 57.51, 12.32)
Overall *p* value			0.248		0.253		0.378
Per 5 cigarettes/day^d^	20,545	543	1.04 (0.96, 1.13)	264	1.04 (0.93, 1.17)	21,088	− 5.34 (− 13.68, 3.00)
Pack-years							
Never	11,035	310	1.00 (reference)	153	1.00 (reference)	11,345	0.00 (reference)
< 15 pack-years^d^	4258	110	0.95 (0.75, 1.21)	55	0.99 (0.71, 1.38)	4368	− 3.22 (− 29.42, 22.99)
≥ 15 pack-years^d^	5252	123	0.90 (0.71, 1.15)	56	0.87 (0.61, 1.24)	5375	− 25.33 (− 62.08, 1.42)
Overall *p* value			0.709		0.728		0.156
Per 5 pack-years^d^	20,545	543	1.00 (0.95, 1.05)	264	1.00 (0.93, 1.08)	21,088	− 2.38 (− 7.26, 2.49)

ORs adjusted for age, sex, centre, education level, physical activity level, BMI, alcohol consumption, coffee intake, and red and processed meat intake

Total *n* = 25,387 (quantified smoking [cigarettes/day, pack‑years] data were available for a subset of ever‑smokers [*n* = 21,088])

^a^Cut-off for positivity ≥ 65 units/ml

^b^Cut-off for high positivity ≥ 167.5 units/ml

^c^Continuous variable of GAD65Ab levels. No cut-off level for positivity

^d^Former or current smoker

### Prospective analyses of incident diabetes in relation to baseline smoking, GAD65Ab levels and T1D-GRS

Having GAD65Ab at baseline was associated with a higher hazard of developing diabetes (HR 1.83, 95% CI 1.65, 2.02), and this was even higher in those with high GAD65Ab positivity (HR 2.97, 95% CI 2.59, 3.39) (ESM Table 3). The T1D-GRS was not associated with incident diabetes, except among those who were GAD65Ab positive (HR per SD increase 1.45, 95% CI 1.27, 1.66) and high GAD65Ab positive (HR per SD increase 1.63, 95% CI 1.34, 1.98) (ESM Table 4).

Current vs never smoking was associated with a higher hazard of diabetes among individuals who were GAD65Ab negative (HR 1.40, 95% CI 1.34, 1.48), GAD65Ab positive (HR 1.40, 95% CI 1.03, 1.90) and high GAD65Ab positive (HR 2.21, 95% CI 1.33, 3.66) (Table 2). The rate of diabetes increased with the number of pack-years in individuals who were GAD65Ab negative (HR per 5 pack-years: 1.05, 95% CI 1.04, 1.06), GAD65Ab positive (HR 1.13, 95% CI 1.02, 1.26) and high GAD65Ab positive (HR 2.02, 95% CI 1.34, 3.03) (Table 2).

**Table 2:** HRs (95% CIs) of incident diabetes in relation to smoking status by baseline GAD65Ab status

	GAD65Ab negative		GAD65Ab positive^a^		High GAD65Ab positive^b^	
	Non-cases/cases, *n*	HR (95% CI)	Non-cases/cases, *n*	HR (95% CI)	Non-cases/cases, *n*	HR (95% CI)
Smoking status						
Never	6610/4425	1.00 (reference)	141/169	1.00 (reference)	54/99	1.00 (reference)
Former	3764/3353	1.22 (1.16, 1.28)	68/111	1.29 (0.96, 1.74)	28/64	1.57 (0.97, 2.53)
Current	3576/2993	1.40 (1.34, 1.48)	67/110	1.40 (1.03, 1.90)	24/60	2.21 (1.33, 3.66)
Overall *p* value		< 0.001		0.068		0.006
Never	6610/4425	1.00 (reference)	141/169	1.00 (reference)	54/99	1.00 (reference)
Ever^c^	7340/6346	1.30 (1.25, 1.36)	135/221	1.34 (1.04, 1.73)	52/124	1.83 (1.22, 2.74)
Cigarettes/day						
Never	6610/4425	1.00 (reference)	141/169	1.00 (reference)	54/99	1.00 (reference)
< 20 cigarettes/day^c^	4090/3103	1.30 (1.23, 1.37)	63/107	1.72 (1.21, 2.44)	22/58	1.62 (0.92, 2.83)
≥ 20 cigarettes/day^c^	1061/1256	1.56 (1.45, 1.68)	22/41	1.68 (1.04, 2.73)	9/22	2.71 (1.04, 7.05)
Overall *p* value		< 0.001		0.006		0.070
Per 5 cigarettes/day^c^	11,761/8784	1.07 (1.05, 1.09)	226/317	1.06 (0.90, 1.26)	85/179	2.22 (1.37, 3.59)
Pack-years						
Never	6610/4425	1.00 (reference)	141/169	1.00 (reference)	54/99	1.00 (reference)
< 15 pack-years^c^	2666/1592	1.16 (1.09, 1.24)	49/61	1.51 (1.01, 2.24)	18/37	1.36 (0.74, 2.50)
≥ 15 pack-years^c^	2485/2767	1.54 (1.45, 1.63)	36/87	1.94 (1.31, 2.89)	13/43	2.78 (1.35, 5.74)
Overall *p* value		< 0.001		0.003		0.018
Per 5 pack-years^c^	11,761/8784	1.05 (1.04, 1.06)	226/317	1.13 (1.02, 1.26)	85/179	2.02 (1.34, 3.03)

HRs adjusted for age (underlying timescale), centre (stratified baseline hazard), sex, education level, physical activity level, BMI, alcohol consumption, coffee intake, and red and processed meat intake

Total *n* = 25,387 (cigarettes/day and pack-years available for *n* = 21,088)

^a^Cut-off for positivity ≥ 65 units/ml

^b^Cut-off for high positivity ≥ 167.5 units/ml

^c^Former or current smoker

### Prospective analyses of incident diabetes in relation to interactions between baseline smoking, GAD65Ab status and T1D-GRS

**Fig. 1:**
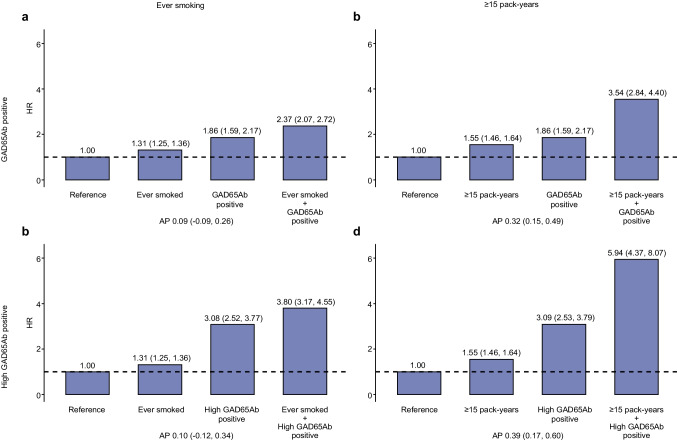
Two-way interaction plots of smoking and GAD65Ab positivity and diabetes incidence. HRs (95% CIs) of incident diabetes in relation to smoking and GAD65Ab status, alone and in combination: (**a**) ever smoking and being GAD65Ab positive (*p* for multiplicative interaction: 0.807, HR_MI_ 0.97 [0.79, 1.20]), (**b**) ≥ 15 pack-years smoking and being GAD65Ab positive (*p* for multiplicative interaction: 0.131, HR_MI_ 1.23 [0.94, 1.60]), (**c**) ever smoking and being high GAD65Ab positive (*p* for multiplicative interaction: 0.673, HR_MI_ 0.94 [0.72, 1.24]), and (**d**) ≥ 15 pack-years smoking and being high GAD65Ab positive (*p* for multiplicative interaction: 0.247, HR_MI_1.24 [0.86, 1.79]). HRs adjusted for age (underlying timescale), centre (stratified baseline hazard), sex, education level, physical activity level, BMI, alcohol consumption, coffee intake, and red and processed meat intake. Reference group is the combination of never smoked and being GAD65Ab negative. Total *n* = 25,387 (pack-years available for *n* = 21,088). Smoking refers to former or current smoking. Cut-off for GAD65Ab positivity was ≥ 65 units/ml and high positivity was ≥ 167.5 units/ml. Additive interaction was estimated as AP (95% CI), calculated from HRs on the multiplicative scale, which reflects the proportion of cases attributable to the interaction. The *p* values, obtained from the interaction term in models with product terms for smoking and GAD65Ab status, test for multiplicative interaction; *p* < 0.05 indicates that the combined effect differs from the product of the effect of each exposure considered alone. An HR_MI_ > 1 indicates that the observed risk in doubly exposed individuals exceeds the expected multiplicative effect, whereas an HR_MI_ < 1 indicates that the observed risk is lower than expected under multiplicativity

The combination of being GAD65Ab positive and smoking conferred a higher HR of incident diabetes than either individual exposure and there was evidence of additive interaction between long-term heavy smoking (≥ 15 pack-years) and being GAD65Ab positive (combined HR 3.54, 95% CI 2.84, 4.40; AP 0.32, 95% CI 0.15, 0.49) (Fig. 1, ESM Table 5). Individuals who were high GAD65Ab positive and long-term heavy smokers had the highest hazard (HR 5.94, 95% CI 4.37, 8.07) (Fig. 1d). Additive interactions were observed between being high GAD65Ab positive and heavy smoking (≥ 20 cigarettes/day, AP 0.33, 95% CI 0.02, 0.64) and long-term heavy smoking (≥ 15 pack-years, AP 0.39, 95% CI 0.17, 0.60) (Fig. 1, ESM Table 6), but there was no evidence of multiplicative interaction. Additionally, there was an additive interaction between low GAD65Ab positivity and heavy smoking (≥ 15 pack-years, AP 0.32, 95% CI 0.09, 0.55) (ESM Table 7).

There was no evidence of an interaction between ever smoking, high T1D-GRS and being GAD65Ab positive (Table 3, ESM Fig. 3). Combined exposure to heavy smoking (≥ 20 cigarettes/day), a high T1D-GRS and being GAD65Ab positive conferred a HR of 12.30 (95% CI 7.23, 20.92) compared with being unexposed to all three factors and there was additive (AP 0.74, 95% CI 0.58, 0.91) and multiplicative (*p* = 0.001) interaction between the three exposures. Additive interaction was also observed between long-term heavy (≥ 15 pack-years) smoking, high T1D-GRS and being GAD65Ab positive (combined HR 7.30, 95% CI 5.17, 10.31, AP 0.46, 95% 0.23, 0.70) (Table 3, ESM Fig. 3b).

**Table 3:** Three-way interaction between smoking, high T1D-GRS and being GAD65Ab positive with regard to the incidence of diabetes, assessed on the additive and multiplicative scale

	T1D-GRS^a^	GAD65Ab^b^	Non-cases/cases, *n*	HR (95% CI)	Additive interaction	Multiplicative interaction	
					AP (95% CI)	*p* value	HR_MI_ (95% CI)
Smoking status							
Never	Low	Negative	3463/2383	1.00 (reference)			
Ever^c^	Low	Negative	4092/3540	1.26 (1.19, 1.33)			
Never	High	Negative	1824/1168	0.94 (0.87, 1.01)			
Ever^c^	High	Negative	1922/1716	1.38 (1.29, 1.47)			
Never	Low	Positive	67/54	1.44 (1.10, 1.89)			
Ever^c^	Low	Positive	68/72	1.44 (1.14, 1.83)			
Never	High	Positive	41/89	3.35 (2.70, 4.15)			
Ever^c^	High	Positive	44/102	3.32 (2.71, 4.06)	− 0.06 (− 0.40, 0.27)	0.489	0.85 (0.53, 1.35)
Cigarettes/day							
Never	Low	Negative	3463/2383	1.00 (reference)			
≥ 20 cigarettes/day^c^	Low	Negative	576/709	1.58 (1.44, 1.74)			
Never	High	Negative	1824/1168	0.93 (0.87, 1.00)			
≥ 20 cigarettes/day^c^	High	Negative	290/307	1.39 (1.22, 1.59)			
Never	Low	Positive	67/54	1.42 (1.08, 1.86)			
≥ 20 cigarettes/day^c^	Low	Positive	14/12	1.27 (0.71, 2.26)			
Never	High	Positive	41/89	3.43 (2.76, 4.26)			
≥ 20 cigarettes/day^c^	High	Positive	4/16	12.30 (7.23, 20.92)	0.74 (0.58, 0.91)	0.001	4.24 (1.78, 10.1)
Pack-years							
Never	Low	Negative	3463/2383	1.00 (reference)			
≥ 15 pack-years^c^	Low	Negative	1440/1606	1.47 (1.37, 1.58)			
Never	High	Negative	1824/1168	0.92 (0.86, 0.99)			
≥ 15 pack-years^c^	High	Negative	626/705	1.55 (1.41, 1.70)			
Never	Low	Positive	67/54	1.43 (1.09, 1.88)			
≥ 15 pack-years^c^	Low	Positive	24/32	1.93 (1.34, 2.76)			
Never	High	Positive	41/89	3.27 (2.63, 4.06)			
≥ 15 pack-years^c^	High	Positive	6/34	7.30 (5.17, 10.31)	0.46 (0.23, 0.70)	0.237	1.45 (0.78, 2.67)

HRs adjusted for age (underlying time scale), centre (stratified baseline hazard), sex, education level, physical activity level, BMI, alcohol consumption, coffee intake, and red and processed meat intake

Total *n* = 20,645 (cigarettes/day and pack-years available for *n* = 21,088; genetic data available for 81.3% of participants)

T1D-GRS is the sum of risk alleles from type 1 diabetes-associated SNPs (*n* = 28) and haplotypes (*n* = 5), weighted by log_*e*_(OR) and averaged per allele. Additive interaction was estimated as AP (95% CI). The *p* values, obtained from the interaction term in models with product terms for smoking and GAD65Ab status, test for multiplicative interaction; *p* < 0.05 indicates that the combined effect differs from the product of the effect for each exposure considered alone. For multiplicative interaction, an HR_MI_ > 1 indicates that the observed risk in doubly exposed individuals exceeds the expected multiplicative effect, whereas an HR_MI_ < 1 indicates that the observed risk is lower than expected under multiplicativity

^a^Cut-off between high and low was at the highest tertile of T1D-GRS

^b^Cut-off for positivity ≥ 65 units/ml

^c^Former or current smoker

### Sensitivity analyses

The increased risk of adult-onset diabetes in relation to being GAD65 positive at baseline, ever smoking and their combination with a high T1D-GRS persisted in sensitivity analyses additionally adjusted for family history of type 2 diabetes, waist circumference, and tea, vegetable, fruit or fish intake or excluding individuals who potentially had undiagnosed diabetes at baseline (ESM Figs. 4–7). Similarly, the lack of association between smoking status (never vs former or current smoking) and being GAD65Ab positive at baseline remained across sensitivity analyses (ESM Fig. 8).

## Discussion

Our findings indicate that smoking is not associated with GAD65 autoimmunity but instead exacerbates the increased risk of diabetes associated with being GAD65Ab positive, especially in the case of long-term heavy smoking. While smoking was associated with a similar hazard of diabetes in GAD65Ab-negative and GAD65Ab-positive individuals, higher hazards were observed in individuals with high GAD65Ab levels. Moreover, the three-way interaction between being GAD65Ab positive, heavy smoking and a high T1D-GRS indicates that genetic susceptibility further intensifies the adverse effects of smoking and autoimmunity on diabetes risk. These results raise the hypothesis that refraining from smoking in individuals with autoimmune markers or a genetic predisposition to type 1 diabetes may mitigate diabetes risk.

Our findings align with those of previous studies observing an increased risk of adult-onset type 1 diabetes [7], LADA [6] and type 2 diabetes [4] in smokers. This contrasts with findings in children of mothers who smoked during pregnancy, where a reduced risk of childhood type 1 diabetes has been observed [8]. This may reflect a protective effect restricted to prenatal exposure or differences in disease mechanisms between children and adults. To our knowledge, this is the first study to investigate smoking as a potential trigger of diabetes-related autoantibodies. We show that smokers are no more likely to be GAD65Ab positive than non-smokers, suggesting that smoking does not initiate the autoimmune process. Instead, we provide novel insight that smoking seems to exacerbate the already increased diabetes risk conferred by such positivity, with the greatest impact observed in individuals with high GAD65Ab levels, which is indicative of a more pronounced autoimmune process. We cannot rule out the possibility that smoking may trigger other diabetes-related autoantibodies, such as antibodies against insulinoma-associated protein 2 (IA-2A), zinc transporter type 8 (ZnT8A) and insulin (IAA), which were not assessed in this study. However, it is worth noting that GAD65Ab is by far the most common autoantibody observed in adults developing diabetes [31]. Regarding potential mechanisms linking smoking to type 2 diabetes, nicotine, as the main active substance in cigarettes, has been suggested to be the causal driver [5]. This is supported by observations of smokers being more insulin resistant than non-smokers [32]. The nicotine mechanism of action is complex and may include induced lipolysis and the release of NEFAs, with reduced insulin sensitivity as a consequence [33], while also promoting a proinflammatory state that may contribute to the autoimmune processes involved in diabetes progression [34]. Moreover, insulin resistance may itself induce progression of autoimmune diabetes through excessive beta cell demand, subsequently causing pancreatic function decline [35]. Indeed, human studies have identified insulin resistance as a predictor for type 1 diabetes [36] and LADA [37], which are both characterised by autoimmune pathogenesis. Taken together, the combination of more severe insulin resistance and beta cell stress from nicotine exposure may cause a higher rate of beta cell decline and a significantly elevated risk of diabetes progression in smokers, particularly heavy smokers.

We observed that heavy smoking accentuated diabetes risk in individuals with both genetic predisposition to type 1 diabetes and markers of autoimmunity. This is in line with findings in LADA, where smoking in combination with high-risk HLA genotypes has been shown to interact on risk [6]. As demonstrated here and previously [2], genetic susceptibility was associated with higher GAD65Ab levels while also increasing the risk in GAD65Ab-positive individuals, suggesting it may intensify the autoimmune response. This is supported by studies linking genetic susceptibility to type 1 diabetes to an increase in antibody-producing cells [38] and multiple islet autoimmunity [39]. HLA DR/DQ haplotypes, which are high-risk factors for type 1 diabetes, contributed a significant proportion to the T1D-GRS of this study [20, 40]. The HLA DR/DQ molecules have various functions in the immune system, including generating suppressive regulatory T cells while also presenting antigens to inflammatory T cells [41]. Carrying multiple high-risk HLA DR/DQ variants may drive a stronger, unregulated autoimmune response, causing more severe immune damage [40]. Potentially, the pronounced insulin resistance in smokers sensitises beta cells to immune damage [42], which is aggravated in those with a genetic predisposition to autoimmunity. This combination could cause a higher rate of beta cell loss, accelerating diabetes progression in GAD65Ab carriers.

This is the first study to investigate the association between smoking and diabetes risk in GAD65Ab-positive individuals. The prospective case–cohort design effectively reduces recall bias and reverse causation. As a multinational study, it provides a uniquely large and diverse sample, which allows for adjustment for various confounding variables. Further, the collection of biological samples and GAD65Ab measurements in such a large cohort is a unique strength of this study and enabled the novel investigation of smoking and autoimmunity and diabetes risk. In addition, GAD65Ab levels were measured at one laboratory in Seattle, minimising measurement variability.

This study also has some limitations. Autoantibody status data were unavailable at diagnosis, making it impossible to confirm when participants first developed GAD65Ab positivity or whether those who had tested positive for GAD65Ab at baseline continued to be positive at the time of diagnosis and, thus, satisfied LADA criteria. Previous studies indicate that approximately 10% of clinically diagnosed type 2 diabetes cases may be misclassified autoimmune diabetes cases [43]. Loss of autoimmunity may have occurred in some participants, while others may have developed GAD65Ab positivity during follow‑up. However, GAD65Ab positivity has been shown to remain stable in most individuals [44]. As repeat testing was not available, some false-positive GAD65Ab results cannot be excluded, although this is unlikely among individuals with high antibody titres. In our cohort, participants with high GAD65Ab levels and those with both GAD65Ab positivity and a high T1D-GRS are most likely to represent true autoimmune diabetes. These groups showed the strongest association with smoking, supporting the validity of our findings. Collection of smoking status information and GAD65Ab measurement at baseline restricted analyses to a cross-sectional assessment of their relationship, and thus temporality could not be evaluated. However, the absence of an association between being GAD65Ab positive at baseline and former smoking, an exposure presumed to have occurred before GAD65Ab study measurements, implies that smoking likely does not act as a trigger for autoimmunity. Additionally, as participants were not aware of their GAD65Ab status at the time of reporting smoking status, the risk of recall bias is minimal.

Smoking status was self-reported, which may have led to some exposure misclassification; however, validation studies comparing questionnaire data with serum cotinine levels have shown high concordance [45]. Given the prospective design, any misclassification of exposure is likely to be non-differential and would therefore bias the effect estimates towards the null. Nonetheless, there is a possibility that early-stage diabetes symptoms manifesting long before the time of diagnosis and enrolment could have promoted smoking cessation in some cases. Alternatively, differential outcome misclassification could have occurred if non-smokers, inclined towards seeking medical care due to their overall healthier lifestyle, were more likely to receive a diagnosis. However, both scenarios imply that cases would be less likely to smoke, which would lead to underestimation of the association with smoking. Misclassification of diagnosis may be due to some identified cases being prevalent cases with delayed diagnosis at baseline. However, this seems unlikely, as sensitivity analysis showed no apparent influence on the association between smoking and diabetes when excluding those with HbA_1c_ ≥ 48 mmol/mol (≥ 6.5%) at baseline and those diagnosed within 2 years of the start of the study. We also acknowledge that other T1D-GRS may perform better in predicting type 1 diabetes [46]. However, the score used in this study has been validated in populations from Europe [20] and North America [47] and was selected to maintain consistency with previous analyses in this cohort [2].

The small sample sizes in the GAD65Ab-positive groups may have reduced point estimate precision, as reflected by the wider 95% CIs compared with GAD65Ab-negative groups. While non-overlapping 95% CIs between GAD65Ab-negative and GAD65Ab-positive individuals who smoked suggest differences in the association between smoking and diabetes across GAD65Ab strata, caution is warranted when interpreting the findings.

We observed interaction mainly on the additive scale rather than the multiplicative scale. Additive interaction is more relevant from a public health perspective, as it assesses the number of people for whom the disease would be prevented if only one of the factors (e.g. being GAD65Ab positive but not a smoker) was present, assuming that all exposures are causative. The combination of both additive and multiplicative interaction has been regarded as the strongest evidence of interaction [48]. This was only observed between heavy smoking, high T1D-GRS and GAD65Ab positivity and therefore our findings should be interpreted with caution.

This study demonstrates that smoking increases diabetes risk in adults irrespective of GAD65 autoimmunity, but may add to the elevated risk associated with such autoimmunity. The risk is greater when combined with genetic susceptibility to type 1 diabetes, highlighting that GAD65Ab carriers with a genetic predisposition for type 1 diabetes are particularly vulnerable. AP estimation indicated that nearly 50% of diabetes cases in those with long-term heavy smoking, GAD65 autoimmunity and type 1 diabetes genetic susceptibility could be potentially prevented by avoiding smoking throughout their lifetime. However, the finding that smoking intensity and pack-years were linearly associated with diabetes risk suggests a potential benefit of quitting at any stage.

As this study used data from European countries its findings are generalisable to populations of European descent and may not generalise to other populations. Phenotypic differences exist across ethnicities in both type 1 and type 2 diabetes, reflecting variation in clinical presentation, progression, genetic factors and environmental exposures [49], including smoking [50]. Thus, similar studies in other populations are warranted. Sex-specific analyses were not conducted due to limited power within strata of GAD65Ab positivity, and the findings reflect average effects across sexes. Additionally, further research is needed to explore the mechanistic role of nicotine as the primary causal factor. With the growing popularity of alternative nicotine products, studies examining their impact on diabetes risk could provide valuable insights into nicotine’s role while also identifying any risks associated with their use. This study also highlights the importance of considering interactions between multiple risk factors, suggesting that future research should explore other potential risk factor interactions.

## Conclusion

Our findings indicate that, although smoking may not be related to the production of autoantibodies, it is associated with an increased risk of progressing into overt diabetes in individuals with GAD65 autoimmunity and a genetic predisposition to type 1 diabetes. The combination of these factors may augment the risk conferred by heavy smoking, suggesting a potential interaction.

## Supplementary Information

Below is the link to the electronic supplementary material.

Supplementary file1 (PDF 1.76 MB)

## Data Availability

The datasets analysed in the current study are available on request to the EPIC steering committee of the MRC Epidemiology Unit.

## Abbreviations

AP: Attributable proportion due to interaction
GAD65Ab: Glutamic acid decarboxylase 65 kDa isoform autoantibody
HLA: Human leukocyte antigen
HR: Hazard ratio
LADA: Latent autoimmune diabetes in adults
OR: Odds ratio
T1D-GRS: Type 1 diabetes genetic risk score
