# Supplementary material for: Smoking, GAD65 autoimmunity, genetic susceptibility to type 1 diabetes and incident adult-onset diabetes in the EPIC-InterAct case–cohort study
Source: Metabologia. 2026 Apr 16;2(1):2. doi: 10.1007/s44357-026-00003-9 (PMC13131207; doi:10.1007/s44357-026-00003-9)
Supplement: Supplementary file 1 — Supplementary file1 (PDF 1.76 MB) [file 44357_2026_3_MOESM1_ESM.pdf]

ELECTRONIC SUPPLEMENTARY MATERIAL

Smoking, GAD65 autoimmunity, genetic susceptibility to type 1 diabetes and incident adult-onset diabetes in the EPIC-InterAct case–cohort study

Authors:

Emmy Keysendal[1], Sofia Carlsson[1], Anna-Maria Lampousi[1], Christiane S. Hampe[2], José María Huerta[3,4], Nicola Kerrison[5], Peter M. Nilsson[6], Valeria Pala[7], Matthias B. Schulze [8,9], Stephen J. Sharp[5], Olov Rolandsson[10], Nicholas Wareham[5]

[1]Institute of Environmental Medicine, Karolinska Institutet, Stockholm, Sweden

[2]Department of Medicine, University of Washington School of Medicine, Seattle, WA, U SA

[3]Department of Epidemiology, Murcia Regional Health Council-IMIB, Murcia, Spain

[4]CIBER Epidemiología y Salud Pública (CIBERESP), Madrid, Spain

[5]Medical Research Council Epidemiology Unit, Institute of Metabolic Science, University of Cambridge, Cambridge, UK

[6]Department of Clinical Sciences, Clinical Research Center, Skåne University Hospital, Lund University, Malmö, Sweden

[7]Epidemiology and Prevention Unit, Fondazione IRCCS Istituto Nazionale dei Tumori, Milan, Italy

[8]Department of Molecular Epidemiology, German Institute of Human Nutrition Potsdam-Rehbruecke, Nuthetal, Germany

[9]Institute of Nutritional Science, University of Potsdam, Potsdam, Germany

[10]Department of Public Health and Clinical Medicine, Family Medicine, Umeå University, Umeå, Sweden

ESM Table 1 Baseline characteristics by GAD65Ab status among all eligible subcohort participants and incident diabetes cases.

	**Subcohort**			**Incident diabetes cases**		
**Characteristic**	**GAD65Ab negative**	**GAD65Ab positive**	***p*-value[a]**	**GAD65Ab negative**	**GAD65Ab positive**	***p*-value[b]**
Individuals, *n*	14623[c]	299[d]		10771	390	
Follow-up, years	12.3 (11.1-13.3)	12.3 (11.1-13.2)	0.607	6.9 (4.4-9.5)	6.4 (3.8-9.3)	0.05
Age, years	52.3 (9.1)	52.7 (9.3)	0.431	55.5 (7.6)	54.9 (8.4)	0.245
Female sex	62.4	64.9	0.420	50.0	58.5	0.001
Family history of type 2 diabetes[e]	18.9	13.4	0.116	36.3	31.1	0.144
BMI, kg/m^2	26.0 (4.2)	25.8 (4.2)	0.359	29.7 (4.7)	28.4 (5.1)	<0.001
Waist circumference[f], cm	86.3 (12.6)	86.2 (12.7)	0.848	97.7 (4.7)	93.8 (13.8)	<0.001
High education	20.8	20.4	0.327	13.3	13.8	0.119
Smokers	25.8	23.7	0.544	27.8	28.2	0.509
Physically active	20.1	19.7	0.595	16.8	18.4	0.115
Alcohol consumption, g/day	6.4 (0.9-18.4)	6.0 (1.5-17.9)	0.641	6.2 (0.6-20.4)	5.4 (0.6-15.2)	0.05
Coffee consumption, g/day	261.3 (90-525)	300 (90.7-577.8)	0.489	270.5 (86-525.2)	285.2 (90-500)	0.871
Red and processed meat intake[g], g/day	74.4 (46.6-108.6)	75.1 (46.4-113.0)	0.626	84.5 (53.9-121.7)	73.9 (46.2-103.8)	<0.001

The data is presented as mean (SD), median (IQR), or percentage. Total *n*=25,387, incident cases in subcohort n=696. GAD65Ab measurements truncated at 1000 units/mL.
[a]Comparing GAD65Ab-positive and GAD65Ab-negative subcohort participants.
[b]Comparing GAD65Ab-positive and GAD65Ab-negative diabetes incidence cases.
[c]4.6% incident diabetes cases during follow-up.
[d]7.7% incident diabetes cases during follow-up.
[e]Information of family history available for 49.3% of the participants.
[f]Waist circumference measurements available for 93.2% of the participants.
[g]Total intake of red-and processed meat.

ESM Table 2 ORs and regression coefficients (95% CI) of baseline GAD65Ab positivity and high GAD65Ab positivity by T1D-GRS.

**T1D-GRS**	**GAD65Ab negative, *n***	**GAD65Ab positive[a]**		**GAD65Ab positive-high[b]**	
		n	**OR (95% CI)**	***n***	**OR (95% CI)**
Low	6701	115	1.00 (reference)	49	1.00 (reference)
Intermediate	6777	146	1.25 (0.97, 1.60)	55	1.15 (0.78, 1.69)
High	6630	276	2.42 (1.94, 3.02)	165	3.39 (2.46, 4.67)
per SD	20645	537	1.66 (1.51, 1.83)	269	2.06 (1.80, 2.36)

ORs adjusted for age and sex, stratified by centre. GRS for type 1 diabetes (T1D-GRS) is the sum of risk alleles from type 1 diabetes-associated SNPs (*n*=28) and haplotypes (*n*=5), weighted by $\log_e$(OR) and averaged per allele. Standard deviation and tertiles of T1D-GRS were assessed according to the distribution in the subcohort. Total *n*=20,645.

[a]Cut-off for positive was ≥65 units/mL, compared to GAD65Ab negative participants.

[b]Cut-off for high-positive was ≥167.5 units/mL, compared to GAD65Ab negative participants

ESM Table 3 HRs (95% CI) of incident diabetes in relation to baseline GAD65Ab status.

**GAD65Ab**	**Non-cases/cases, *n***	**HR (95% CI)**
Negative	13950/10771	1.00 (reference)
Positive ≥65 units/ml	276/390	1.83 (1.65, 2.02)
Positive-low 65 to <167.5 units/ml	170/167	1.20 (1.03, 1.40)
Positive-high ≥167.5 units/ml	106/223	2.97 (2.59, 3.39)

HRs adjusted for age (underlying time scale), centre (stratified baseline hazard), sex, education level, physical activity level, BMI, alcohol consumption, coffee intake, and red and processed meat intake.
Total *n*=25,387.

ESM Table 4 HRs (95% CI) of incident diabetes in relation to T1D-GRS by baseline GAD65Ab status.

T1D-GRS	Full cohort		GAD65Ab negative		GAD65Ab positive[b]		GAD65Ab positive-high[c]	
	**Non-cases/cases, *n***	**HR (95% CI)**	**Non-cases/cases, *n***	**HR (95% CI)**	**Non-cases/cases, *n***	**HR (95% CI)**	**Non-cases/cases, *n***	**HR (95% CI)**
Low[a]	7690/6049	1.00 (reference)	7555/5923	1.00 (reference)	135/126	1.00 (reference)	48/56	1.00 (reference)
High[a]	3831/3075	1.02 (0.97, 1.06)	3746/2884	0.98 (0.94, 1.03)	85/191	2.04 (1.57, 2.65)	36/129	2.28 (1.53, 3.41)
per SD	11521/9124	1.02 (1.00, 1.04)	11301/8807	1.00 (0.98, 1.02)	220/317	1.45 (1.27, 1.66)	84/185	1.63 (1.34, 1.98)

HRs adjusted for age (underlying time scale), sex and centre (stratified baseline hazard). Total *n*=25,387.

GRS for type 1 diabetes is the sum of risk alleles from type 1 diabetes-associated SNPs (*n*=28) and haplotypes (*n*=5), weighted by $\log_e$(OR) and averaged per allele. Standard deviation and tertiles of T1D-GRS were assessed according to the distribution in the subcohort.

[a]Cut-off level between high and low at highest tertile of T1D-GRS

[b]Cut-off for positive was ≥65 units/mL, compared to GAD65Ab-negative participants.

[c]Cut-off for high-positive was ≥167.5 units/mL, compared to GAD65Ab-negative participants.

ESM Table 5 Two-way interaction between being GAD65Ab positive and smoking with regard to the incidence of diabetes, assessed on the additive and multiplicative scale.

				Additive interaction	Multiplicative interaction	
		Non-cases/cases, *n*	HR (95% CI)	AP (95% CI)	*p*-value	HR_{MI} (95% CI)
**Smoking status**	***GAD65Ab***[a]					
Never	Negative	6610/4425	1.00 (reference)			
Ever[b]	Negative	7340/6346	1.31 (1.25, 1.36)			
Never	Positive	141/169	1.86 (1.59, 2.17)			
Ever[b]	Positive	135/221	2.37 (2.07, 2.72)	0.09 (-0.09, 0.26)	0.807	0.97 (0.79, 1.20)
**Cigarettes per day**	***GAD65Ab***[a]					
Never	Negative	6610/4425	1.00 (reference)			
<20cigarettes/day[b]	Negative	4090/3103	1.30 (1.23, 1.37)			
≥20cigarettes/day[b]	Negative	1061/1256	1.57 (1.46, 1.69)			
Never	Positive	141/169	1.86 (1.59, 2.17)			
<20cigarettes/day[b]	Positive	63/107	3.44 (2.83, 4.19)			
≥20cigarettes/day[b]	Positive	22/41	2.80 (2.05, 3.83)	0.13 (-0.16, 0.42)	0.819	0.96 (0.68, 1.36)
**Pack-years**	***GAD65Ab***[a]					
Never	Negative	6610/4425	1.00 (reference)			
<15 pack-years[b]	Negative	2666/1592	1.16 (1.09, 1.24)			
≥15 pack-years[b]	Negative	2485/2767	1.55 (1.46, 1.64)			
Never	Positive	141/169	1.86 (1.59, 2.17)			
<15 pack-years[b]	Positive	49/61	2.94 (2.27, 3.80)			
≥15 pack-years[b]	Positive	36/87	3.54 (2.84, 4.40)	0.32 (0.15, 0.49)	0.131	1.23 (0.94, 1.60)

HRs adjusted for age (underlying time scale), centre (stratified baseline hazard), sex, education level, physical activity level, BMI, alcohol consumption, coffee intake, and red- and processed meat intake. Total *n*=25,387 (cigarettes/day and pack-years available for *n*=21,088). Additive interaction was estimated as AP (95% CI). The *p*-values, obtained from the interaction term in models with product terms for smoking and GAD65Ab status, test for multiplicative interaction; $p<0.05$ indicates that the combined effect differs from the product of the effect for each exposure considered alone. For multiplicative interaction, an $HR_{MI} >1$ indicates that the observed risk in doubly exposed individuals exceeds the expected multiplicative effect, whereas an $HR_{MI} <1$ indicates that the observed risk is lower than expected under multiplicativity.

[a]Cut-off for GAD65Ab positivity was ≥65 units/mL and high positivity was ≥167.5 units/mL.

[b]Former or current smoker.

ESM Table 6 Two-way interaction between being high GAD65Ab positive and smoking with regard to the incidence of diabetes, assessed on the additive and multiplicative scale.

				Additive interaction	Multiplicative interaction	
		Non-cases/cases, *n*	HR (95% CI)	AP (95% CI)	*p*-value	HR_{MI} (95% CI)
**Smoking status**	**GAD65Ab**[a]					
Never	Negative	6610/4425	1.00 (reference)			
Ever[b]	Negative	7340/6346	1.31 (1.25, 1.36)			
Never	Positive-high	54/99	3.08 (2.52, 3.77)			
Ever[b]	Positive-high	52/124	3.80 (3.17, 4.55)	0.11 (-0.12, 0.34)	0.673	0.94 (0.72, 1.24)
**Cigarettes per day**	**GAD65Ab**[a]					
Never	Negative	6610/4425	1.00 (reference)			
<20cigarettes/day[b]	Negative	4090/3103	1.30 (1.23, 1.37)			
≥20cigarettes/day[b]	Negative	1061/1256	1.57 (1.46, 1.68)			
Never	Positive-high	54/99	3.10 (2.53, 3.80)			
<20cigarettes/day[b]	Positive-high	22/58	4.24 (3.26, 5.52)			
≥20cigarettes/day[b]	Positive-high	9/22	5.45 (3.55, 8.35)	0.33 (0.02, 0.64)	0.640	1.12 (0.70, 1.80)
**Pack-years**	**GAD65Ab**[a]					
Never	Negative	6610/4425	1.00 (reference)			
<15 pack-years[b]	Negative	2666/1592	1.16 (1.09, 1.24)			
≥15 pack-years[b]	Negative	2485/2767	1.55 (1.46, 1.64)			
Never	Positive-high	54/99	3.09 (2.53, 3.79)			
<15 pack-years[b]	Positive-high	18/37	3.59 (2.59, 4.98)			
≥15 pack-years[b]	Positive-high	13/43	5.94 (4.37, 8.07)	0.39 (0.17, 0.60)	0.247	1.24 (0.86, 1.79)

HRs adjusted for age (underlying time scale), centre (stratified baseline hazard), sex, education level, physical activity level, BMI, alcohol consumption, coffee intake, and red- and processed meat intake (g/day). Total n=25,050 (cigarettes/day and pack-years available for n=20,809). Additive interaction was estimated as AP (95% CI). The p-values and HR_{MI}, obtained from the interaction term in models with product terms for smoking and GAD65Ab status, test for multiplicative interaction; $p<0.05$ indicates that the combined effect differs from the product of the effect for each exposure considered alone. For multiplicative interaction, an HR_{MI} >1 indicates that the observed risk in doubly exposed individuals exceeds the expected multiplicative effect, whereas an HR_{MI} <1 indicates that the observed risk is lower than expected under multiplicativity.

[a]Cut-off for GAD65Ab positivity was ≥65 units/mL and high positivity was ≥167.5 units/mL.

[b]Former or current smoker.

ESM Table 7 Two-way interaction between being low GAD65Ab positive and smoking with regard to the incidence of diabetes, assessed on the additive and multiplicative scale.

				Additive interaction	Multiplicative interaction	
		Non-cases/cases, *n*	HR (95% CI)	AP (95% CI)	*p*-value	HR_{MI} (95% CI)
**Smoking status**	**GAD65Ab[a]**					
Never	Negative	6610/4425	1.00 (reference)			
Ever[b]	Negative	7340/6346	1.30 (1.25, 1.36)			
Never	Positive-low	87/70	1.19 (0.94, 1.51)			
Ever[b]	Positive-low	83/97	1.59 (1.30, 1.95)	0.06 (-0.20, 0.32)	0.875	1.03 (0.75, 1.40)
**Cigarettes per day**	**GAD65Ab[a]**					
Never	Negative	6610/4425	1.00 (reference)			
<20cigarettes/day[b]	Negative	4090/3103	1.30 (1.23, 1.37)			
≥20cigarettes/day[b]	Negative	1061/1256	1.56 (1.45, 1.68)			
Never	Positive-low	87/70	1.19 (0.94, 1.51)			
<20cigarettes/day[b]	Positive-low	41/49	2.85 (2.14, 3.80)			
≥20cigarettes/day[b]	Positive-low	13/19	1.77 (1.12, 2.79)	0.01 (-0.46, 0.49)	0.860	0.95 (0.57, 1.60)
**Pack-years**	**GAD65Ab[a]**					
Never	Negative	6610/4425	1.00 (reference)			
<15 pack-years[b]	Negative	2666/1592	1.16 (1.09, 1.24)			
≥15 pack-years[b]	Negative	2485/2767	1.54 (1.45, 1.63)			
Never	Positive-low	87/70	1.19 (0.94, 1.51)			
<15 pack-years[b]	Positive-low	31/24	2.31 (1.54, 3.45)			
≥15 pack-years[b]	Positive-low	23/44	2.54 (1.88, 3.44)	0.32 (0.09, 0.55)	0.097	1.39 (0.94, 2.03)

HRs adjusted for age (underlying time scale), centre (stratified baseline hazard), sex, education level, physical activity level, BMI, alcohol consumption, coffee intake, and red- and processed meat intake (g/day). Total *n*=25,058 (cigarettes/day and pack-years available for *n*=20,824). Additive interaction was estimated as AP (95% CI). The *p*-values, obtained from the interaction term in models with product terms for smoking and GAD65Ab status, tests for multiplicative interaction; $p<0.05$ indicates that the combined effect differs from the product of the effect for each exposure considered alone. For multiplicative interaction, an HR_{MI} >1 indicates that the observed risk in doubly exposed individuals exceeds the expected multiplicative effect, whereas an HR_{MI} <1 indicates that the observed risk is lower than expected under multiplicativity.

[a]Low GAD65Ab positive defined as ≥65–<167.5 units/mL.

[b]Former or current smoker.

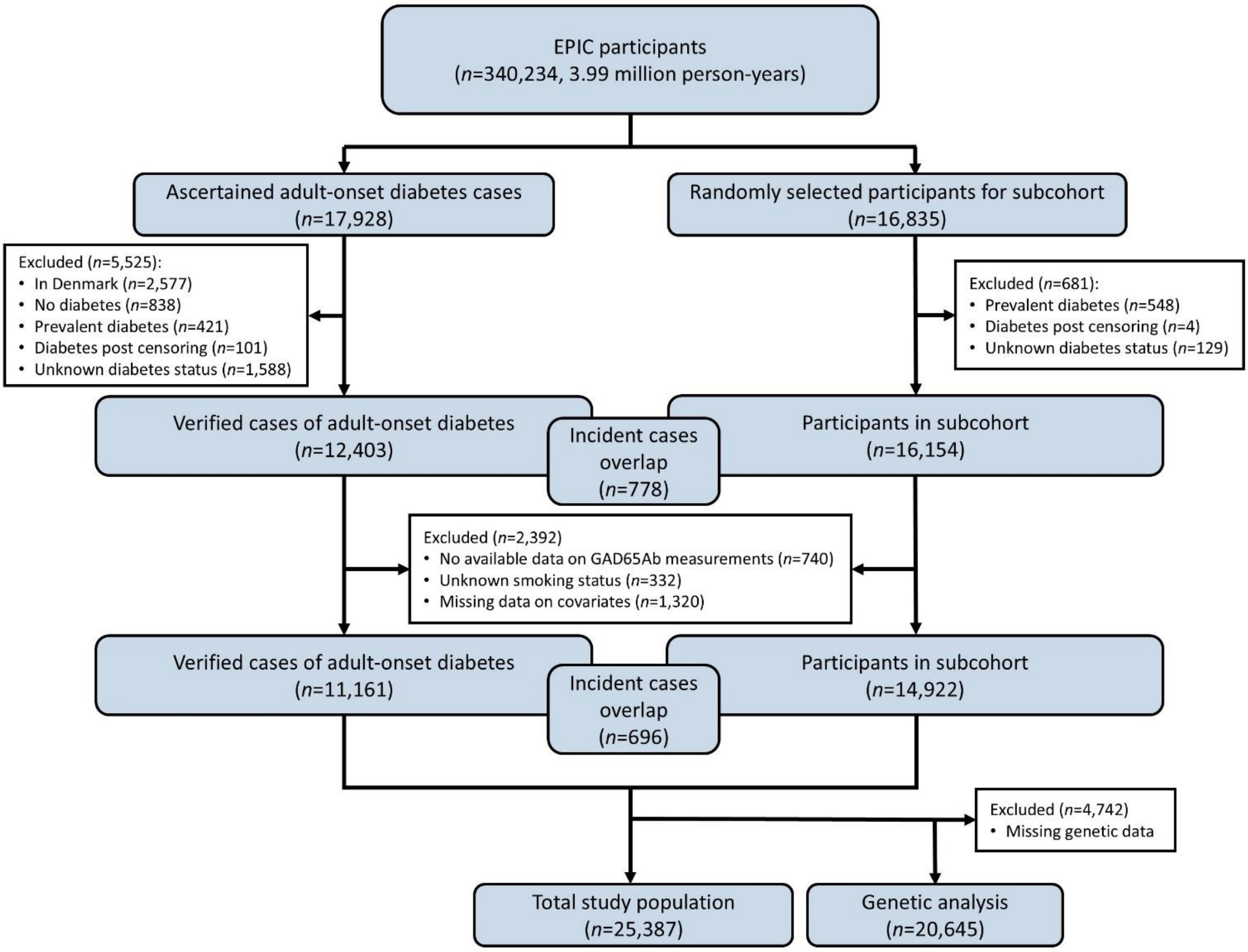

ESM Figure 1 Flowchart of study participants in the EPIC-InterAct case-cohort study.

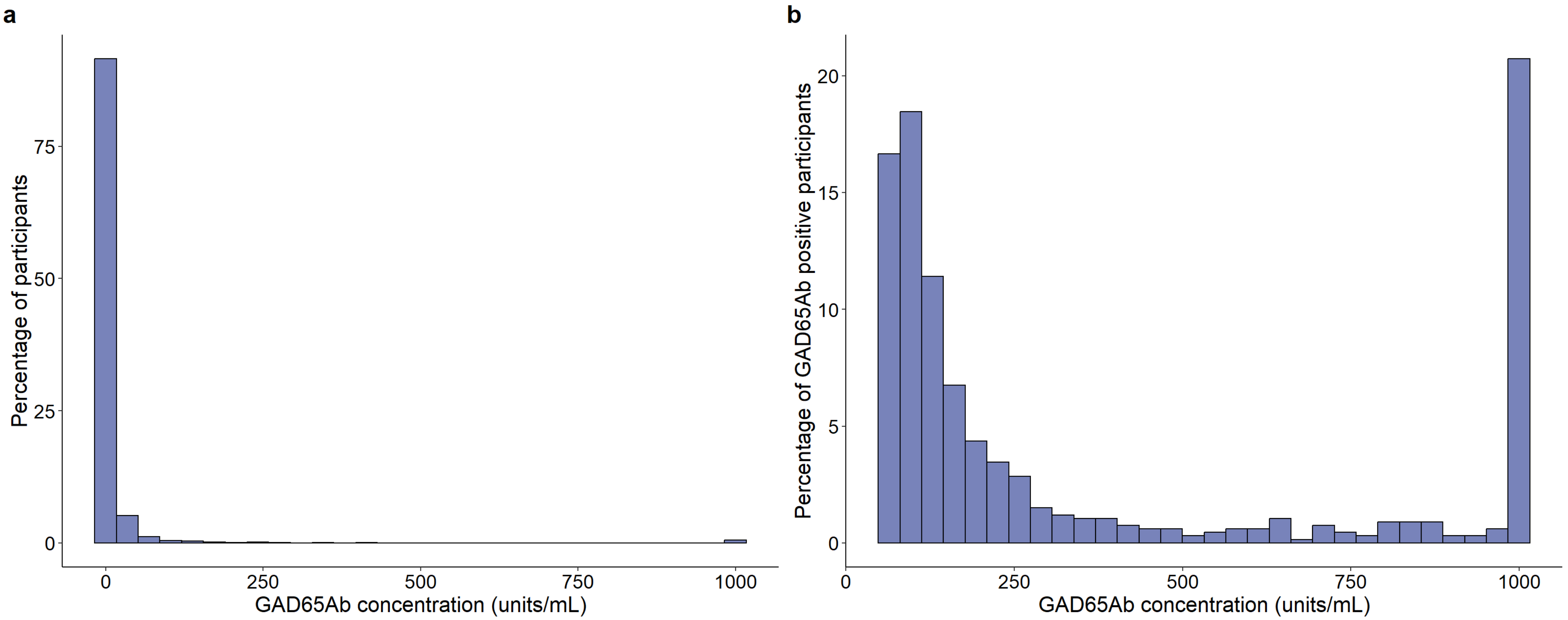

ESM Figure 2 Distribution of GAD65Ab among **(a)** all study participants (*n*=25,387), among **(b)** GAD65Ab-positive (≥65 units/mL, *n*=666).

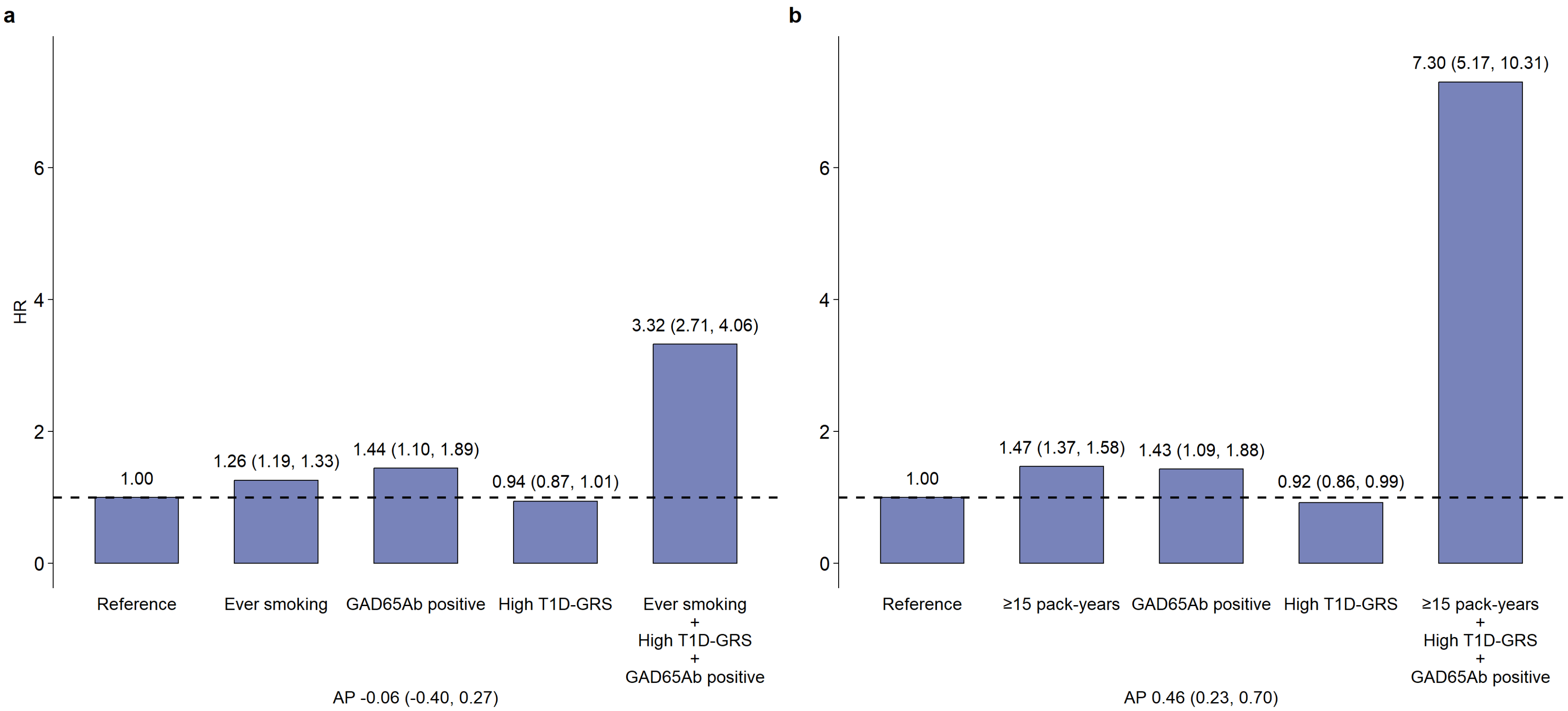

ESM Figure 3 Three-way interaction plots of smoking, GAD65Ab positivity, high T1D-GRS and diabetes incidence. HRs (95% CIs) of incident diabetes in relation smoking, GAD65Ab status, and T1D-GRS, alone and in combination: **(a)** ever smoking, being GAD65Ab positive, and high T1D-GRS (*p* for multiplicative interaction: 0.489, HR_{MI} 0.85 [0.53, 1.35]), **(b)** ≥15 pack-years smoking, being GAD65Ab positive, and high T1D-GRS (*p* for multiplicative interaction: 0.237, HR_{MI} 1.45 [0.78, 2.67]. HRs adjusted for age (underlying time scale), centre (stratified baseline hazard), sex, education level, physical activity level, BMI, alcohol consumption, coffee intake, and red- and processed meat intake. Reference group is the combination of never smoked, being GAD65Ab negative, and low T1D-GRS. Total *n*=20,645 (pack-years available for *n*=21,088, genetic data available for 81.3% of participants). Smoking refers to former or current smoking. Cut-off for GAD65Ab positivity was ≥65 units/mL and high positivity was ≥167.5 units/mL. Cut-off level between high and low at the highest tertile of T1D-GRS. Additive interaction was estimated as AP (95% CI), calculated from HR on the multiplicative scale, which reflects the proportion of cases attributable to the interaction. The *p*-values, obtained from the interaction term in models with product terms for smoking, GAD65Ab status, and T1D-GRS, test for multiplicative interaction; $p<0.05$ indicates that the combined effect differs from the product of the effect for each exposure considered alone. For multiplicative interaction, an HR_{MI} >1 indicates that the observed risk in doubly exposed individuals exceeds the expected multiplicative effect, whereas an HR_{MI} <1 indicates that the observed risk is lower than expected under multiplicativity.

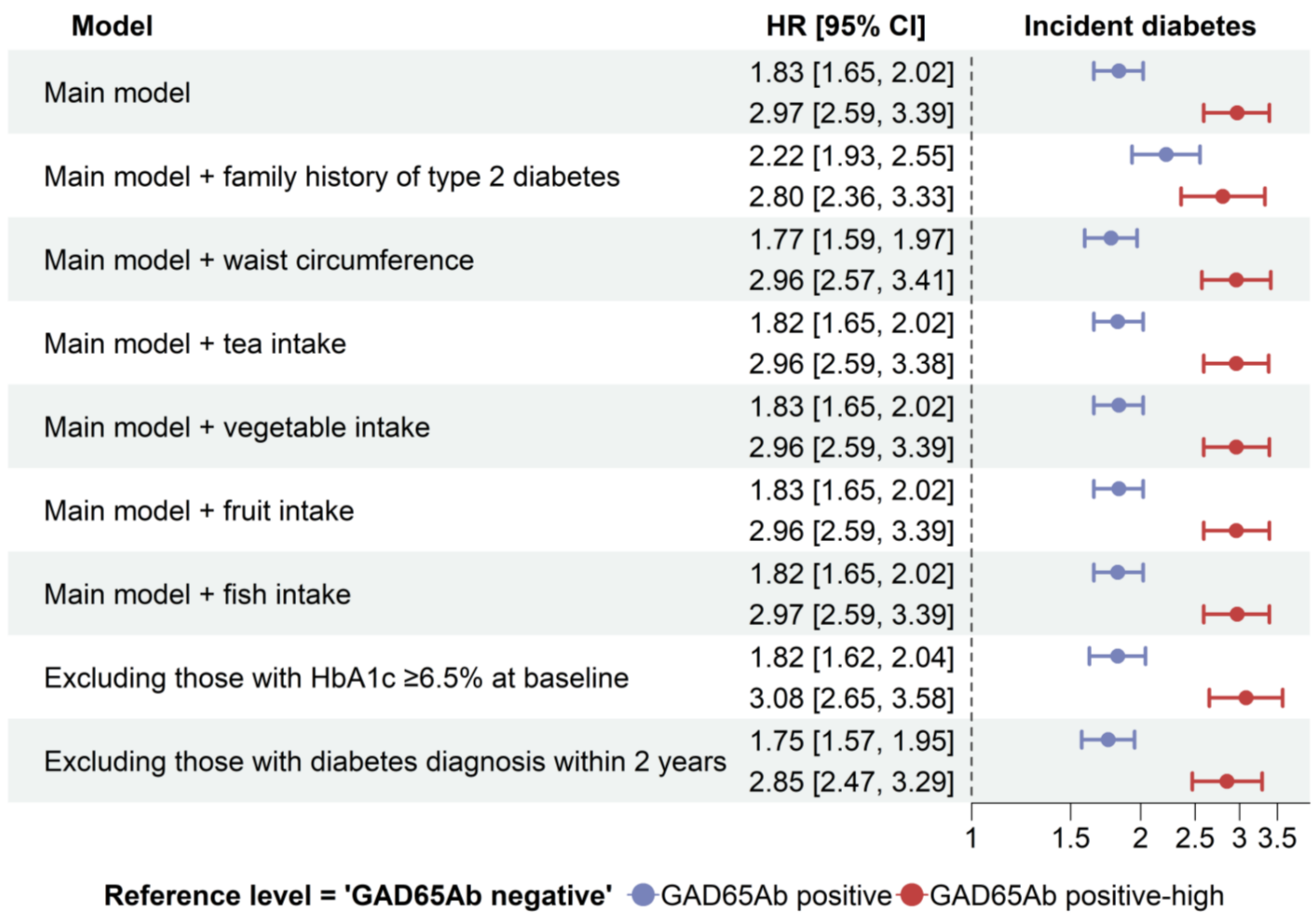

ESM Figure 4 HRs (95% CI) of incident diabetes in relation to GAD65Ab positivity, categorised as GAD65Ab positive (≥65 units/mL) and GAD65Ab positive-high (≥167 units/mL) vs GAD65Ab negative, based on different sensitivity analyses. The main model is adjusted for age (underlying time scale), centre (stratified baseline hazard), sex, education level, physical activity level, BMI, intake of alcohol, coffee, and red- and processed meat. Waist circumference measurements available for 93.2% of participants, family history information available for 49.3% of participants, excluded individuals with HbA1c≥6.5% *n*=2,118, excluded individuals with diabetes diagnosis within 2 years *n*=950.

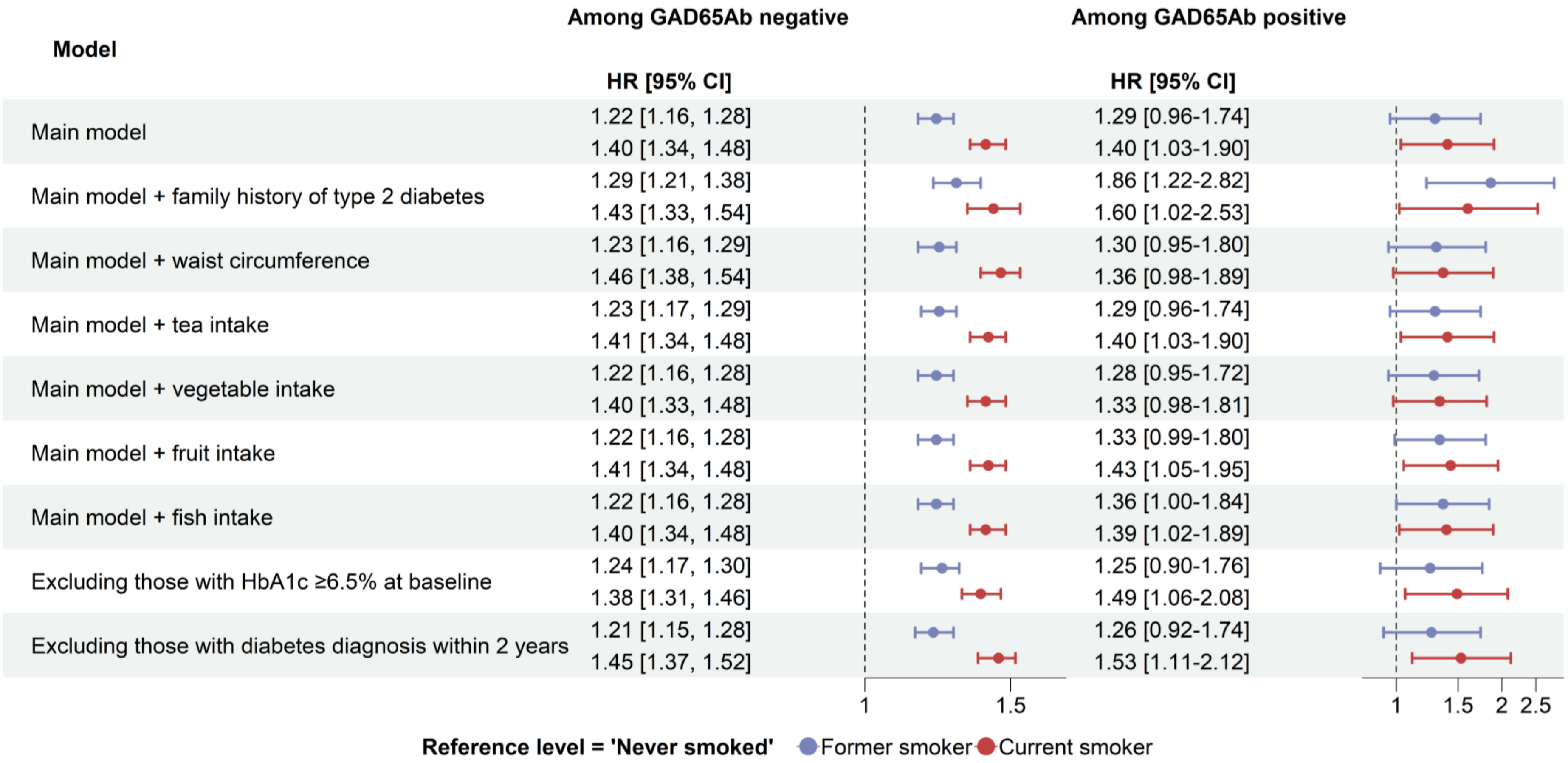

ESM Figure 5 HRs (95% CI) of incident diabetes in relation to former smoking or current smoking vs never smoking among GAD65Ab-negative individuals (n= 24,721) and GAD65Ab-positive individuals (≥65 units/mL, n=666), based on different sensitivity analyses. The main model is adjusted for age (underlying time scale), centre (stratified baseline hazard), sex, education level, physical activity level, BMI, intake of alcohol, coffee, and red and processed meat. Waist circumference measurements available for 93.2% of participants, family history information available for 49.3% of participants, excluded individuals with HbA1c≥6.5% n=2,118, excluded individuals with diabetes diagnosis within 2 years n=950.

Two-way interaction: GAD65Ab positivity and ever smoking

Model	HR [95% CI]
Main model	2.37 [2.07, 2.72]
Main model + family history of type 2 diabetes	2.97 [2.47, 3.57]
Main model + waist circumference	2.38 [2.06, 2.75]
Main model + tea intake	2.37 [2.07, 2.72]
Main model + vegetable intake	2.37 [2.06, 2.72]
Main model + fruit intake	2.37 [2.07, 2.72]
Main model + fish intake	2.36 [2.06, 2.71]
Excluding those with HbA1c ≥6.5% at baseline	2.40 [2.06, 2.80]
Excluding those with diabetes diagnosis within 2 years	2.28 [1.97, 2.64]

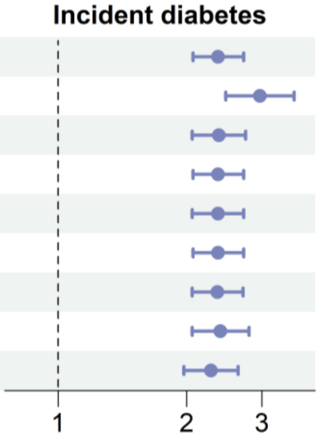

ESM Figure 6 HRs (95% CI) of incident diabetes for the combination of GAD65Ab positivity (≥65 units/mL) and ever smoking (former smoking or current smoking) vs being unexposed to both factors, based on different sensitivity analyses. The main model is adjusted for age (underlying time scale), centre (stratified baseline hazard), sex, education level, physical activity level, BMI, intake of alcohol, coffee, and red- and processed meat. Waist circumference measurements available for 93.2% of participants, family history information available for 49.3% of participants, excluded individuals with HbA1c≥6.5% *n*=2,118, excluded individuals with diabetes diagnosis within 2 years *n*=950.

Three-way interaction: GAD65Ab positivity, high T1D-GRS, and ever smoking

Model	HR [95% CI]
Main model	3.32 [2.71, 4.06]
Main model + family history of type 2 diabetes	3.93 [3.05, 5.06]
Main model + waist circumference	2.82 [2.27, 3.50]
Main model + tea intake	3.33 [2.72, 4.07]
Main model + vegetable intake	3.32 [2.72, 4.07]
Main model + fruit intake	3.32 [2.71, 4.06]
Main model + fish intake	3.31 [2.70, 4.05]
Excluding those with HbA1c ≥6.5% at baseline	3.46 [2.76, 4.34]
Excluding those with diabetes diagnosis within 2 years	3.19 [2.58, 3.95]

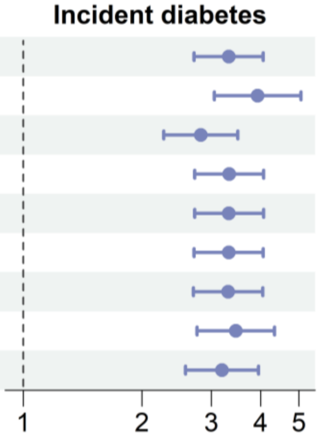

ESM Figure 7 HRs (95% CI) of incident diabetes for the combination of GAD65Ab positivity (≥65 units/mL), ever smoking (former smoking or current smoking) and the highest tertile of a genetic risk score for type 1 diabetes (genetic data available for 77.1% of participants) vs being unexposed to all three factors, based on different sensitivity analyses. The main model is adjusted for age (underlying time scale), centre (stratified baseline hazard), sex, education level, physical activity level, BMI, intake of alcohol, coffee, and red- and processed meat (g/day). Waist circumference measurements available for 92.5 of participants, family history information available for 51.5% of participants, excluded individuals with HbA1c≥6.5% *n*=1,768, excluded individuals with diabetes diagnosis within 2 years *n*=807.

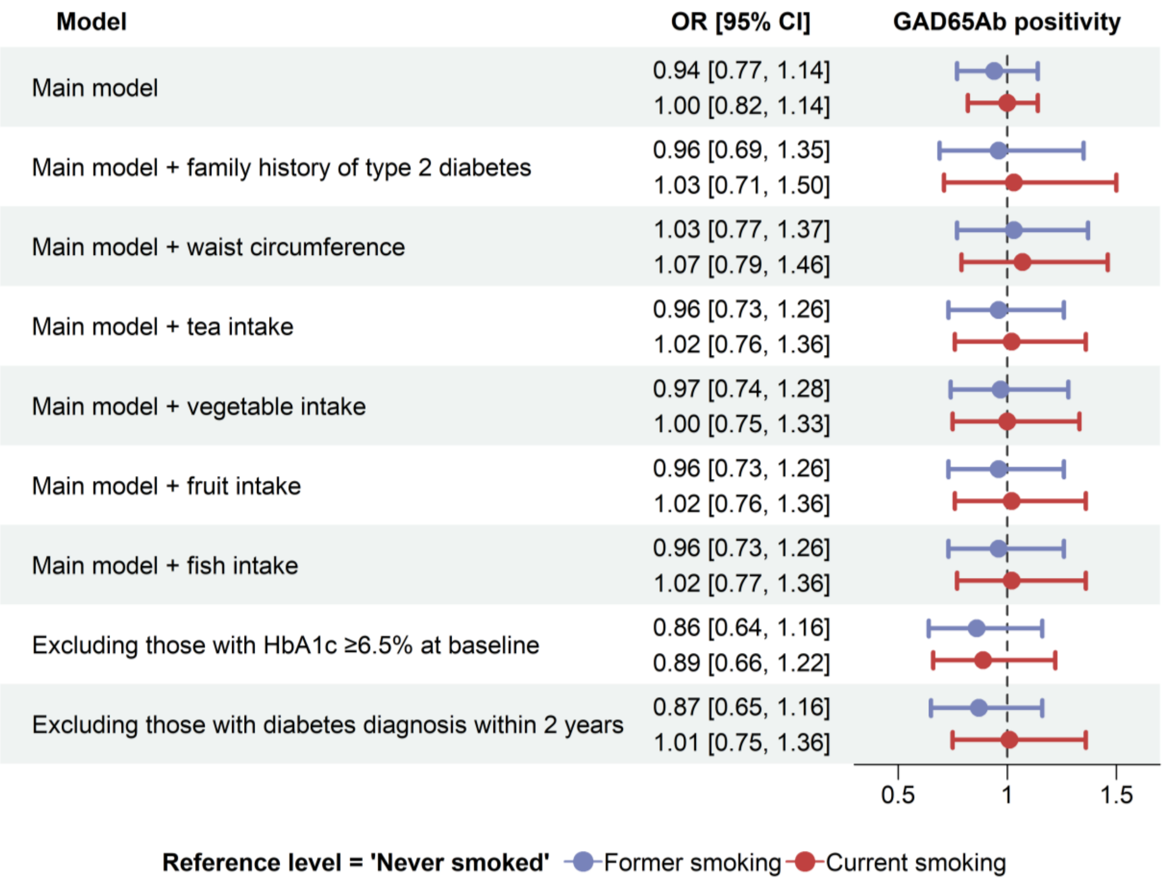

ESM Figure 8 ORs (95% CI) of GAD65Ab positivity in relation to former smoking or current smoking vs never smoking, based on different sensitivity analyses. The main model is adjusted for age, centre, sex, education level, physical activity level, BMI, intake of alcohol, coffee, and red- and processed meat. Waist circumference measurements available for 93.2% of participants, family history information available for 49.3% of participants, excluded individuals with HbA1c≥6.5% *n*=2,118, excluded individuals with diabetes diagnosis within 2 years *n*=950.
